# Self-assembly of promoter DNA and RNA Pol II machinery into transcriptionally active biomolecular condensates

**DOI:** 10.1126/sciadv.adi4565

**Published:** 2023-10-18

**Authors:** Brian A. Lewis, Subhendu Kumar Das, Rajiv Kumar Jha, David Levens

**Affiliations:** Gene Regulation Section, LP/CCR/NCI/NIH, 9000 Rockville Pike, Bethesda, MD 20892, USA.

## Abstract

Transcription in the nucleus occurs in a concentrated, dense environment, and no reasonable biochemical facsimile of this milieu exists. Such a biochemical environment would be important for further understanding transcriptional regulation. We describe here the formation of dense, transcriptionally active bodies in vitro with only nuclear extracts and promoter DNA. These biomolecular condensates (BMCs) are 0.5 to 1 μm in diameter, have a macromolecular density of approximately 100 mg/ml, and are a consequence of a phase transition between promoter DNA and nuclear extract proteins. BMCs are physically associated with transcription as any disruption of one compromised the other. The BMCs contain RNA polymerase II and elongation factors, as well as factors necessary for BMC formation in vivo. We suggest that BMCs are representative of the in vivo nuclear environment and a more physiologically relevant manifestation of the preinitiation complex/elongation machinery.

## Teaser

Transcriptionally active condensates can form de novo with nuclear extracts and a promoter scaffold.

## INTRODUCTION

The intranuclear environment is a crowded one, with protein/DNA/RNA concentrations reaching 100 to 200 mg/ml (*1*, *2*). At these concentrations, the concepts of dilute solution biochemistry are no longer applicable because of the restrictions on the free diffusion space that is reduced both in total volume and in the diffusion paths available (*2*–*4*). Instead, the kinetics of molecular interactions require various models of anomalous diffusion, defined by diffusion in less than three dimensions, to explain behaviors between proteins and enzymes and their various substrates (*5*).

A second consequence of a crowded environment is the emergence of networks of weak interactions (micromolar ranges) between proteins, allowing those weak interactions to manifest themselves. These interactions are mediated by various protein domains, especially low sequence complexity, multivalent, intrinsically disordered regions that elude any sort of classical description of structure (*3*, *6*–*9*). These collections of weak interactions manifest themselves as phase transitions, where a concentrated condensate forms one phase along with a second dilute phase (*10*).

What then is the nature of the nuclear environment? An understanding and recapitulation of the complex cellular and nuclear environments with their nonclassical biochemistry is of paramount importance (*4*, *11*). The existence of such a biochemical environment has been elusive and would be of considerable use in illuminating how such densities contribute to biological functions.

Biochemical experiments have demonstrated protein phase transitions occurring under highly favorable conditions driven by high concentrations of one or perhaps a few recombinant proteins and, in some cases, adding volume excluders to coerce the phase transition. These conditions are likely not representative of the in vivo situation where hundreds or thousands of proteins exist together, each at concentrations much less than the milligrams per milliliter of recombinant proteins used in other studies (*9*, *12*). Clearly, however, many proteins have the capacity to undergo phase transitions, and it is not unreasonable to expect that combinations of them do also.

Data showing that individual transcription-related proteins have domains that can undergo a phase transition to form condensates of liquid droplets and gels have brought to the forefront interactions other than high-affinity “lock and key” interactions (*13*–*16*). The MED1 subunit of mediator, the OCT4 activator, and components of the general transcription machinery have many of the properties expected of a role of transcriptional coactivators in condensate formation (*17*–*19*). It is likely that a spectrum of affinities and interactions exist between different proteins and domains and with specific nucleic acids. Although the weak affinities have been previously dismissed as nonspecific interactions, when acting collectively, hundreds of weak interactions simultaneously become quite relevant in the cramped nuclear milieu. In addition, both DNA and RNA can form condensates with various proteins (*19*–*24*).

We describe here the spontaneous formation of transcriptionally active mesoscale-size bodies/biomolecular condensates (BMCs) in vitro with only nuclear extracts and promoter DNA. The BMCs are 0.5 to 1 μm in diameter. BMCs have properties of condensates: BMC demixing occurs at critical protein and promoter DNA concentrations, while excess protein or DNA returns the system to a single phase. Sucrose density measurements approximate the macromolecular density at 100 mg/ml, in line with in vivo measurements. BMC disruption with KCl or Sarkosyl results in aberrant elongation and a block in early termination, and a catalytic inhibitor of O-GlcNAc transferase (OGT) prevents transcription and demonstrates that BMC formation involves factors beyond the core transcription machinery. Isolation of early elongating BMCs from soluble nuclear extract indicates that they alone support further elongation. These all indicate that the BMC is physically associated with properly functioning transcription. The BMCs contain elongation factors Positive Transcription Elongation Factor b (P-TEFb), Poly-ADP Ribose Polymerase 1 (PARP-1), RNA Polymerase Associated Factor 1 complex (PAF1c), and Host Cell Factor 1 (HCF-1) as well as Non-POU Domain-Containing Octamer binding (NONO), Splicing Factor Proline- and Glutamine-rich (SFPQ), and Fused in Sarcoma (FUS) proteins required for nuclear membrane-less organelle formation in vivo.

## RESULTS

### Cytomegalovirus promoter DNA forms large mesoscale bodies in a nuclear extract

**Fig. 1.**
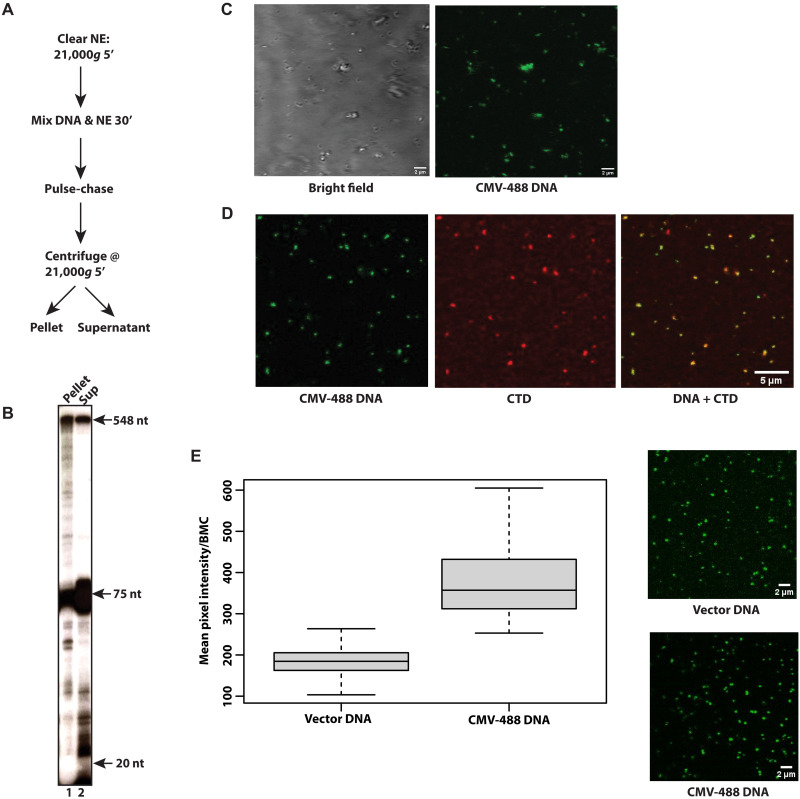
Existence of a transcriptionally active promoter-dependent condensate in a cell-free transcription system. (**A**) Experimental design for (B). (**B**) Detection of a dense RNA product in the cell-free transcription system. In vitro transcription assays were done as in (A). After centrifugation at 15,000 rpm/21,000*g*, the RNA products were isolated and separated on 8% TBE/urea acrylamide gels. Note that the dark band at +75 is tRNA in the extract labeled by the tRNA nucleotidyl transferase (*28*). (**C**) Microscopic imaging of droplets by bright field and Alexa Fluor 488–conjugated CMV promoter. PICs were formed in a standard in vitro transcription reaction and visualized by laser scanning confocal microscopy. (**D**) Promoter DNA and Pol II colocalize in condensates. Bodies were visualized at 488 nm by laser scanning confocal microscopy to detect Alexa Fluor 488–conjugated CMV promoter DNA or with Alexa Fluor 594–conjugated RNA Pol II CTD antibody (8WG16). Images were overlaid with each other or individually with bright-field images. (**E**) Comparison of mesoscale body formation by vector DNA and CMV promoter DNA. Identical amounts of Alexa Fluor 488–conjugated control or CMV promoter DNA were added to nuclear extracts and PICs were assayed by visualization with LSM. Boxplots represent three samples and are the mean pixel intensity of the Alexa Fluor 488 signal per BMC. Below the boxplot is a representative microscopic image.

We and others (*25*–*29*) have previously used HeLa nuclear extracts and the cytomegalovirus (CMV) immediately early promoter in a pulse-chase in vitro transcription assay. If large, transcriptionally active bodies were to form in this system, one might expect that the pulse-chased ^32^P-RNA would be included in a separate, dense phase after a brief microcentrifugation step (Fig. 1A). In vitro transcription assays were assembled by mixing CMV promoter DNA and HeLa nuclear extract together for 30 min, forming the requisite preinitiation complexes (PICs). Transcription was initiated with a 30 s pulse of 0.5 μl of ^32^P-cytidine triphosphate (CTP) (0.1 μM) and 0.5 mM guanine/adenine/uridine triphosphate (GAU), followed by a chase with 3 mM nonradioactive CTP for 5 min. The reactions were centrifuged (15,000 rpm/21,000*g* 5 min), and RNA was isolated from the pellet and supernatant and analyzed by Tris-borate-EDTA/urea polyacrylamide electrophoresis. Quite unexpectedly, approximately 50% of the full-length 548-nucleotide RNA and most of the intermediate length RNAs were recovered in the pellet after centrifugation (Fig. 1B). We also found that most of the transfer RNAs (tRNAs) at +75 are in the supernatant of these spin experiments herein (Fig. 1B and fig. S1B). [Note that the dark band at +75 is tRNA in the extract labeled by endogenous tRNA nucleotidyl transferase and ^32^P-CTP (*28*) and is a typical background event in pulse-chase assays using ^32^P-CTP (*25*, *27*–*31*). This is illustrated in fig. S1A where the tRNA labeling occurs upon the addition of ^32^P-CTP and in the absence of promoter DNA (lane 2). Lane 3 shows that the addition of α-amanitin (2 μg/ml) abrogates RNA polymerase II (Pol II) transcription, as expected at this concentration of inhibitor (*32*). These data show that the tRNA band at +75 is background and is not a product of any promoter-dependent transcription in the pulse-chase assay.]

Such large, dense bodies might also be visually apparent with bright-field microscopy and would show whether meso-body formation was promoter or transcription dependent. Adding CMV-promoter DNA (0.2 pmol) to the nuclear extract formed bodies as revealed by either bright-field or laser scanning confocal microscopy using Alexa Fluor 488–labeled CMV promoter (Fig. 1C). (Note that the nuclear extracts were centrifuged for 5 min at 21,000*g* at 4°C to remove any insoluble materials before using them in either pulse-chase or microscopy experiments.) Bright-field images showed that the bodies were approximately 0.5 to 1 μm in diameter and that both DNA and RNA Pol II [using a Pol II C-terminal domain (CTD) antibody] colocalized within the same bodies (Fig. 1D). We also compared the Alexa Fluor 488–labeled CMV promoter (0.2 pmol) to an Alexa Fluor 488–conjugated vector backbone (0.2 pmol) and found that the PIC-stage bodies formed in a promoter-dependent manner, although there appears to be a general DNA component as well (although no transcriptional activity) (Fig. 1E). As expected for a promoter-less vector, we did not find any transcription activity in the parent pGL2 vector compared to the CMV promoter in the same vector backbone (fig. S1C).

BMC is a term devised to describe a body that is a concentration of biological molecules, in this case, membrane-less (*33*, *34*). The term does not mean to suggest any specific mechanism of condensate formation, other than referring to it as a macromolecular phase separation (*34*). We have chosen then to refer to the bodies described herein as BMCs.

### BMCs are the product of a phase transition between promoter DNA and nuclear proteins

**Fig. 2.**
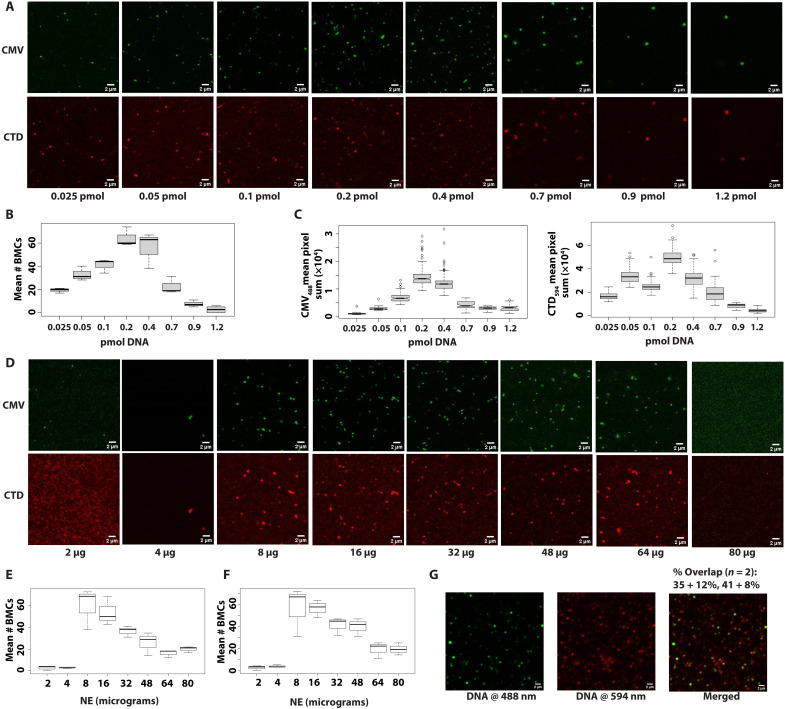
BMCs are a consequence of a phase transition. (**A**) Laser scanning confocal microscopy visualization of the titration of Alexa Fluor 488–CMV promoter DNA (green) into a constant amount of nuclear extract. RNA Pol II was detected using an Alexa Fluor 594–conjugated CTD antibody (red). (**B**) Boxplots of the data in (A), the mean number of BMCs versus CMV promoter [DNA]. (**C**) Boxplots of the data in (A), the partition coefficient (defined as the mean sum of pixels per sample) of CMV Alexa Fluor 488 DNA and CTD Alexa Fluor 594 signals versus CMV promoter concentration. (**D**) Laser scanning confocal microscopy visualization of the titration of HeLa nuclear extract into a constant amount of Alexa Fluor 488–CMV promoter DNA (green; 0.2 pmol). RNA Pol II was detected using an Alexa Fluor 594–conjugated CTD antibody (red). (**E**) Boxplots of (D) show the mean number of Alexa Fluor 488–CMV promoter DNA-containing BMCs formed versus the indicated amount of nuclear extract. (**F**) Boxplots of (D) show the mean number of Alexa Fluor 594–CTD–containing BMCs formed versus the indicated amount of nuclear extract. (**G**) The amount of overlap between equal concentrations of CMV promoter templates PCR-labeled with either Alexa Fluor 488 or Alexa Fluor 594 is shown. The percentage of overlap is the result of two independent experiments.

To define the BMCs more precisely, we titrated increasing amounts of polymerase chain reaction (PCR)–labeled Alexa Fluor 488–labeled promoter DNA versus a constant amount of nuclear extract (40 μg; this is the amount of nuclear extract (NE) used in the transcription assays in Fig. 1). The increasing DNA resulted in an increase in BMCs followed by a plateau at 0.2 to 0.4 pmol of DNA (Fig. 2, A and B). We saw decreased BMC formation with titration of promoter DNA from 0.9 to 1.2 pmol indicating that the promoter excess prevented BMC formation.

A boxplot of the DNA partition coefficient showed a similar distribution: The DNA and extract undergo a phase transition from 0.025 to 0.4 pmol (Fig. 2C). Thereafter, further increasing [DNA] showed a gradual return to nearly one phase by 1.2 pmol of DNA. RNA Pol II behaved similarly, overall showing a similar level of incorporation as the promoter DNA increased from 0.025 to 0.4 pmol before also trending back to a single phase by 1.2 pmol (Fig. 2C).

We next titrated the nuclear extract against a constant amount of CMV promoter DNA (0.2 pmol; this is the concentration used in the transcription assays) and visualized BMCs by either the Alexa Fluor 488 DNA signal or the Alexa Fluor 594–Pol II antibody signal. We observed a sharp transition/demixing phase transition and formation of BMCs at 8 μg of extract, followed by a subsequent plateau and reversal/progressive dissolution of BMCs by 80 μg of extract (Fig. 2, D to F). The analysis of the nuclear extract titration in a transcription assay showed that the transcription activity generally followed the phase separation transition point (fig. S2).

We do not necessarily expect the bulk phase transition to correlate precisely with the RNA yield of the complete reaction. BMC condensation represents PIC formation, an early stage of the transcription cycle, whereas the yield of RNA product reflects progression through the full cycle. The flux of protein components, nucleotides, and cofactors through the full cycle might be expected to modify the steady-state levels and properties of BMCs. Moreover, the final RNA yield will not reflect BMCs that fail to complete the full transcription cycle. Because different stages of transcription may be optimized at slightly different extract concentrations, reflecting the pool of available transcription components, their titration curves may not be fully coincident.

Thus, both the CMV promoter template and NE titration gave similar results: a transition to form BMCs and the subsequent remixing at higher concentrations of either DNA or nuclear extract. The behaviors of the BMCs after either promoter DNA or nuclear extract titrations are evidence of a phase transition.

### The BMCs contain ~2 promoter DNA templates

We next determined the numbers of CMV promoter templates that existed in the BMCs. The idea here is that an equal mixture of Alexa Fluor 488– and Alexa Fluor 594–conjugated CMV templates (0.1 pmol each; 40 μg of extract) would be distinguishable if the BMCs contained only one template: Individual BMCs would be either green (Alexa Fluor 488) or red (Alexa Fluor 594). If the BMCs contained multiple templates, then a more continuous distribution of the two overlapping would be apparent. In 35 to 40% of the BMCs, green and red colocalized (Fig. 2G), while approximately 60% of the BMCs were either green or red. This result suggests a binomial distribution with ~^3^/_4_ of the BMCs bearing two templates (red-red, red-green, and green-green) and the remaining quarter of the BMCs bearing a single template (either green or red). We interpret these results to suggest that there is an upper limit on the number of promoter DNA templates in a BMC.

We conducted a similar experiment using a 1:1 ratio of an Alexa Fluor 488– and Alexa Fluor 594–conjugated RNA Pol II antibody that recognized the N terminus of the large subunit of RNA pol II (RPB1). In contrast to the DNA signal distribution, we found that the green and red signals mostly overlapped across a broad distribution with the total intensity scaling with the area of the BMC (fig. S3); thus, variable numbers of Pol II were associated with BMCs, with Pol II number contributing to overall BMC size.

### BMCs physically support transcription

**Fig. 3.**
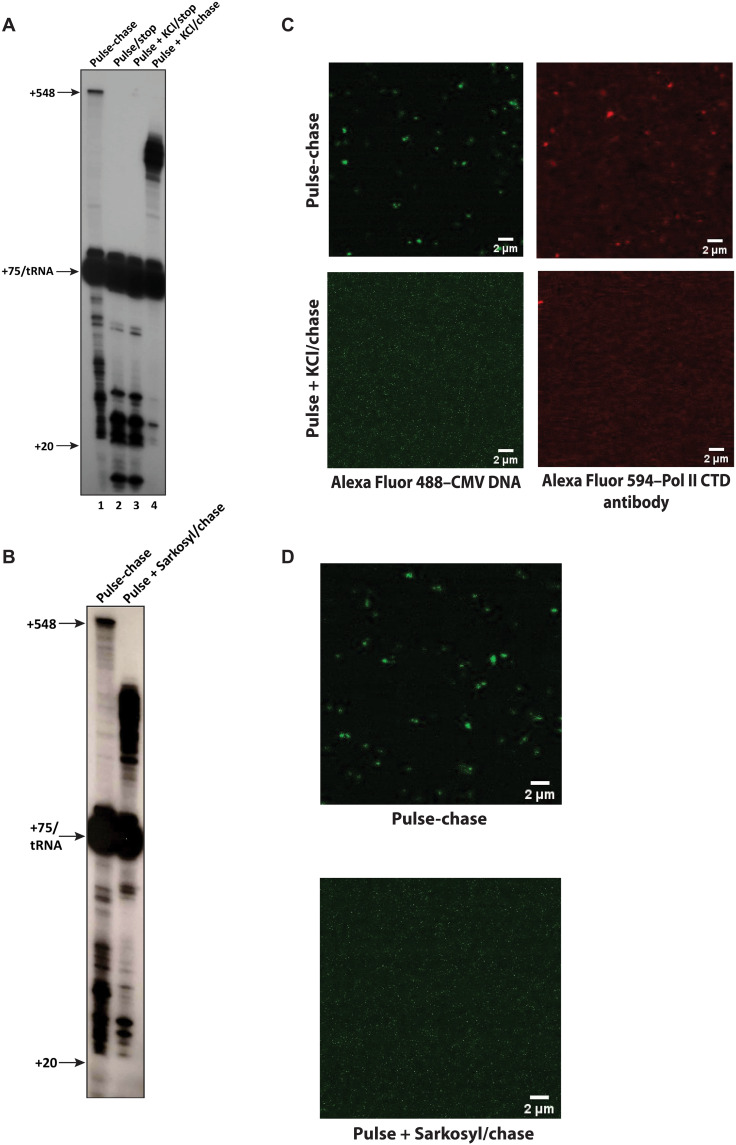
BMCs are functionally coupled with PIC formation and transcriptional elongation. (**A**) KCl (350 mM) disrupts proper elongation in the cell-free transcription system. Lane 1 shows a standard pulse-chase assay. Lane 2 shows a pulse of transcription (^32^P-CTP + GUA) that was immediately stopped with stop buffer. Lane 3 shows a pulse to which KCl was added to 350 mM, mixed, and immediately followed by stop buffer. Lane 4 shows a transcription assay to which a pulse was done for 30 s, followed by addition of KCl to 350 mM final concentration, mixed, and then chased for 5 min with cold CTP before adding stop buffer. The labeled RNA was assessed as in Fig. 1B. (**B**) Sarkosyl disrupts proper elongation in vitro. Lane 1 shows a standard pulse-chase assay. Lane 2 shows a transcription assay to which a pulse was done for 30 s, followed by addition of Sarkosyl to 0.6% final concentration, mixed, and then chased for 5 min with cold CTP before adding stop buffer. The labeled RNA was assessed as in Fig. 1B. (**C**) KCl (350 mM) dissolves BMCs in the cell-free transcription system. Transcription assays representing lanes 1 and 4 in (A) were visualized for Alexa Fluor 488–labeled CMV promoter DNA by confocal microscopy. (**D**) Sarkosyl disrupts BMCs in vitro. Transcription assays representing lanes 1 and 2 in (C) were visualized for Alexa Fluor 488–labeled CMV promoter DNA by confocal microscopy.

If BMCs are required for proper transcription, then disruption of the BMCs should also affect transcription in some manner. We added KCl to 350 mM or Sarkosyl to 0.6% (*35*) to an in vitro transcription assay after the 30 s pulse, followed by a 5 min chase (Fig. 3, A and B). In contrast to the control pulse-chase (Fig. 3, A and B, lane 1), the KCl- and Sarkosyl-treated labeled RNAs below +75, all elongated but aberrantly stopped at ~+200 (Fig. 3A, lane 4; and Fig. 3B, lane 2). Laser scanning confocal microscopy of the KCl- and Sarkosyl-pulsed-transcription reactions showed that both treatments dissolved the BMCs (Fig. 3, C and D), and therefore, the aberrant elongation during the chase step occurred in the one-phase solution. This experiment shows that BMC maintenance is physically coupled with the proper elongation conducted by the nuclear extract.

**Fig. 4.**
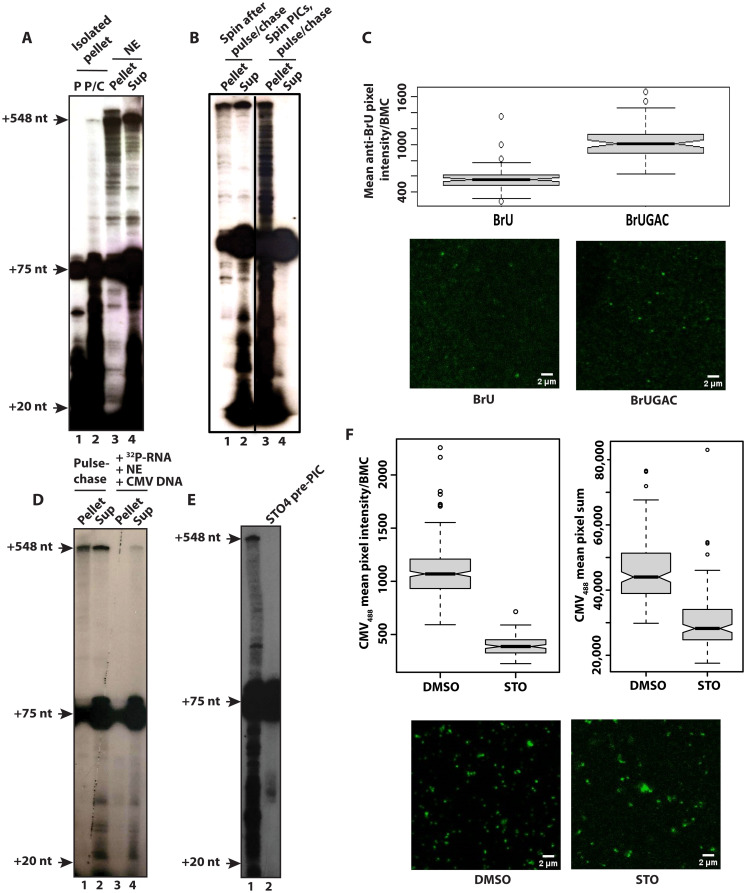
BMCs are transcriptionally active. (**A**) Isolated pulsed Pol II–stage BMCs are transcriptionally active. Pulsed-only in vitro transcriptions were isolated by centrifuging at 15,000 rpm/21,000*g*, washed briefly in and suspended in transcription buffer. The pulsed pellet (lane 1; P) was chased for 5 min with 0.5 mM NTPs (lane 2; P/C). For comparison, lanes 3 and 4 are a complete pulse-chase assay separated into pellet (Pellet) and supernatant fractions (Sup) as in Fig. 1B. (**B**) Pelleted BMCs can synthesize a full-length RNA. PICs were formed with CMV promoter DNA and nuclear extract, centrifuged at 15,000 rpm/21,000*g*, and washed with and resuspended in transcription buffer, followed by a standard pulse-chase assay. (**C**) BMCs to which either BrU or BrUGAC was added were fixed and dried on coverslips and stained with anti-BrU antibody/secondary Alexa Fluor 488 antibody. A boxplot comparing the mean intensity of BrU versus BrUGAC incorporation per BMC, detected by anti-BrU and Alexa Fluor 488 secondary antibodies (*n* = 2) with representative microscopy images is shown. (**D**) Exogenously labeled RNA does not associate with the BMC. ^32^P-labeled RNA was isolated from a standard CMV promoter–driven pulse-chase assay and added to a second transcription reaction at the PIC formation stage. This second reaction was centrifuged; pellet and supernatant were processed, and labeled RNA was assessed as in Fig. 1B. (**E**) OGT inhibitor ST045849 blocks transcription when added at the PIC formation stage. A standard pulse-chase assay, control transcription (lane 1), or with ST045849 (lane 2) is shown. (**F**) ST045849 prevents the formation of functional BMCs when added at the PIC formation stage. Mean intensities/BMC of Alexa Fluor 488–conjugated CMV promoter DNA from one of three separate experiments are shown. The lower graph is a plot of the partition coefficient (defined as the mean sum of pixels per sample) of DNA with dimethyl sulfoxide (DMSO) or ST045849, along with representative microscopy images.

We next did a 30 s pulse in vitro transcription followed by the addition of 20 mM EDTA to stop transcription and found that the pulsed BMCs can be isolated by centrifugation (Fig. 4A, lane 1). We then asked whether these pulse-stopped BMCs could synthesize a full-length RNA after sedimentation. Put another way, do the pulsed BMCs contain the necessary elongation machinery to synthesize a full-length RNA? Pulsed-stopped BMCs were isolated by sedimentation at 15,000 rpm/21,000*g* for 5 min and then rinsed in and resuspended in transcription buffer. After a 5 min chase with cold nucleoside triphosphates (NTPs), the RNA was isolated and visualized by electrophoresis and autoradiography. As can be seen in Fig. 4A, the isolated pulsed RNA BMCs synthesized a full-length RNA after a 5 min chase (compare lanes 1 and 2). These experiments herein show that the BMCs contain factors necessary for productive elongation.

We then asked whether BMCs were transcriptionally active after removal of the dilute phase of the nuclear extract by sedimentation. PICs were assembled for 30 min, then centrifuged to isolate the BMCs, rinsed briefly in and suspended in transcription buffer, followed by a standard pulse-chase assay on both the sedimented BMCs and the separated supernatant (Fig. 4B). RNA analysis showed that the BMCs, even at the PIC stage, could synthesize a full-length RNA product, whereas the resulting supernatant (the dilute phase of the nuclear extract) had no activity (Fig. 4B, lane 4). Transcription by the pelleted and washed BMCs displayed an increased number of intermediate RNA products (Fig. 4B, lane 3), suggesting that components in the dilute phase may augment elongation within the BMC. These experiments show that the pulsed RNA Pol II complexes in the BMCs contain factors necessary for elongation, that transcription is directly associated with the BMCs, and that transcription is not occurring in the soluble supernatant fraction. Last, we formed BMCs with Alexa Fluor 594–CMV DNA, added either bromouridine triphosphate (BrUTP) as a negative control or BrUTP + adenosine 5′-triphosphate (ATP), guanosine 5′-triphosphate (GTP), and CTP (to initiate transcription) for 5 min, fixed the BMCs with paraformaldehyde on coverslips, and immunostained them with anti-BrU antibody. As can be clearly seen, the BrU antibody preferentially stains BMCs in reactions to which all four nucleotides were added (Fig. 4C).

We next examined the possibility that the transcribed RNA bound the BMC after transcription. We first isolated ^32^P-RNA from a standard pulse-chase assay and then added that ^32^P-RNA to a second transcription reaction where PICs had been formed by mixing CMV promoter DNA and nuclear extract. These reactions were then centrifuged, and both pellet and supernatant were processed to isolate RNA. We found that the ^32^P-RNA was in the supernatant (Fig. 4D, lanes 3 and 4), whereas the control pulse-chase showed ^32^P-RNA in the pellet (Fig. 4D, lanes 1 and 2), as in previous experiments herein. This experiment shows that the RNA in the pellet does not arise from soluble RNA adventitiously adhering to and copurifying with the BMC.

We have previously documented the requirement for OGT catalytic activity to form functional PICs and for transcription using nuclear extracts (*36*). Specifically, OGT inhibition hindered RNA Pol II recruitment to PICs (*37*). We further document the OGT inhibitor PIC defect here in Fig. 3I. A pulse-chase assay with the OGT catalytic inhibitor ST045849 (*38*), added concomitantly with nuclear extract and CMV promoter DNA during PIC formation, showed little transcription and no full-length RNAs. We also assessed BMC formation in these reactions. ST045849 markedly disrupted BMC formation as assayed by the observation of Alexa Fluor 488–conjugated CMV promoter DNA (Fig. 4E). These data all show that the BMCs are transcriptionally active, that any disruption of BMCs also abrogates proper transcription initiation and elongation, and last, that the O-GlcNAc posttranslational modification is necessary for BMC formation and transcription.

### BMCs are dense bodies

**Fig. 5.**
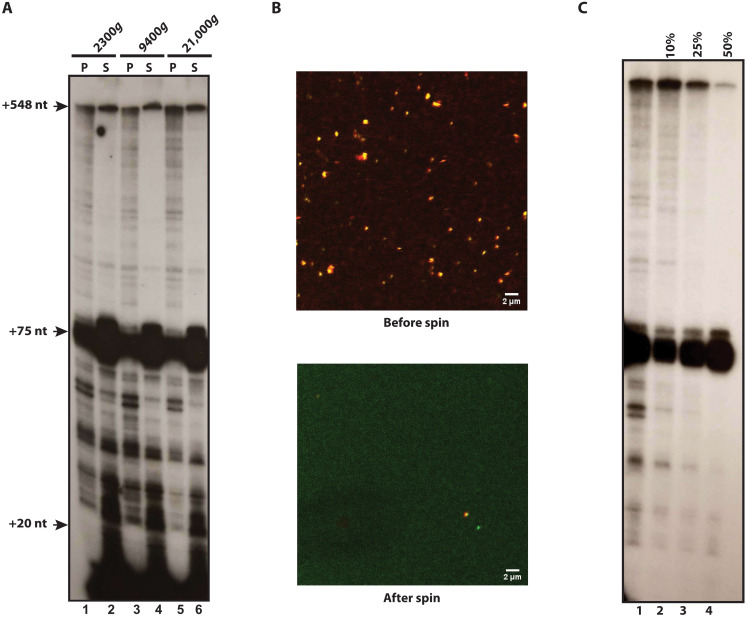
BMCs are dense bodies. (**A**) Low-speed centrifugation pellets labeled RNA. In vitro transcription reactions were centrifuged at the indicated relative centrifugal force (RCF) for 5 min. RNA was isolated from pellet (P) and supernatant (S) and separated as in Fig. 1. (**B**) Condensates were formed with Alexa Fluor 488–CMV promoter followed by a 15,000 rpm/21,000*g* 5 min microfuge spin. Alexa Fluor 594–Pol II CTD antibody was added, and the in vitro transcription reaction was visualized by laser scanning confocal microscopy, before and after centrifugation. The overlap of the 488 and 594 channel images either before or after centrifugation is shown. (**C**) Isolation of BMCs by sucrose cushion density centrifugation using 10, 25, or 50% sucrose. Pellets were processed for ^32^P-RNA, analyzed by urea-TBE 8% polyacrylamide gel, and visualized by autoradiography.

The centrifugation data suggest that the BMCs are very dense. To further examine this property, the BMCs were subjected to several different centrifugation speeds. ^32^P-labeled RNA was isolated from pellets and supernatants and visualized by electrophoresis and autoradiography. Slow centrifugation (5000 rpm/2300*g* for 5 min) sedimented the RNA as effectively as 15,000 rpm/21,000*g* for 5 min (Fig. 5A). We confirmed the removal of BMCs from the transcription reaction by laser scanning confocal microscopy (Fig. 5B). Thus, the BMCs are very large, dense objects.

To assess their density, BMCs were isolated by centrifugation through sucrose cushions. Pulse-chased transcription reactions were placed on top of a 10, 25, or 50% sucrose cushion and spun. The pellets were assayed for labeled RNA by TBE-urea gel electrophoresis (Fig. 5C). The RNA passed through the 10% cushion, was partially hindered by the 25% cushion, and failed to penetrate the 50% cushion (compare lane 1 to lanes 2 and 4; Fig. 5C). The 25% sucrose cushion retains approximately half of the labeled BMCs and thus suggests that the macromolecular density of the BMC is approximately 100 mg/ml, which is 50× the final concentration of protein in the transcription reaction (not accounting for the contribution of the DNA) and which approximates the range of concentrations reported for nuclear compartments in vivo (*1*).

### BMCs contain RNA Pol II elongation- and splicing-associated factors

**Fig. 6.**
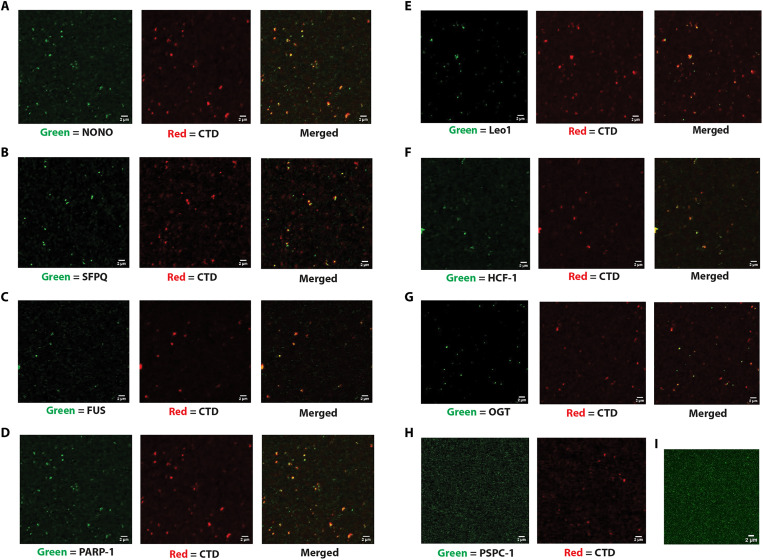
BMCs are complex phases that include NONO, SFPQ, and elongation factors. PICs were formed for 30 min in a standard in vitro transcription assay. The reaction was then mixed with anti–Pol II CTD (red channel), and either anti-NONO (**A**), anti-SFPQ (**B**), anti-FUS (**C**), anti–PARP-1 (**D**), anti-Leo1 (**E**), anti–HCF-1 (**F**), anti-OGT (**G**), anti–PSPC-1 [(**H**); negative control], or rabbit Alexa Fluor 488 secondary [(**I**); negative control] antibodies and visualized with an Alexa Fluor 488 anti-rabbit secondary antibody by laser scanning confocal microscopy. Images of the indicated green-channel (488 nm) antibody overlapped with the red-channel (594 nm) RNA Pol II CTD antibody are shown. Overlaps between the two signals are indicated by orange/yellow hues.

NONO, SFPQ, and FUS are essential for paraspeckle BMC formation in vivo (*39*–*41*). NONO localizes to promoters in vivo genome-wide (*42*), and both NONO and FUS interact with RNA Pol II and splicing factors (*43*–*48*). RNA binding proteins in general have been implicated in condensate formation (*20*). Therefore, we speculated that these proteins might contribute to BMC formation in vitro. We incubated PIC-stage BMCs with antibodies to NONO, SFPQ, and FUS and visualized by laser scanning confocal microscopy using Alexa Fluor 488 anti-rabbit secondary antibodies. RNA Pol II was concomitantly visualized using the Alexa Fluor 594–conjugated CTD antibody 8WG16 (Fig. 6). NONO, SFPQ, and FUS were all readily detected in the BMCs and overlapped extensively with the Pol II signals (middle column and orange/yellow merged images), while the Alexa Fluor 488 secondary antibody alone gave no substantial signal (Fig. 6). In addition, we detected PARP-1 (required for elongation) (*49*), the Leo1 subunit of the PAF complex (elongation) (*50*), HCF-1 (elongation) (*51*, *52*), and OGT (*29*, *36*, *37*) but not the NONO/SFPQ-related protein Paraspeckle Component 1 (PSPC-1) (Fig. 6, C to H). If PSPC-1 interacts with RNA Pol II as previously suggested (*24*), it must do so after PIC formation.

**Fig. 7.**
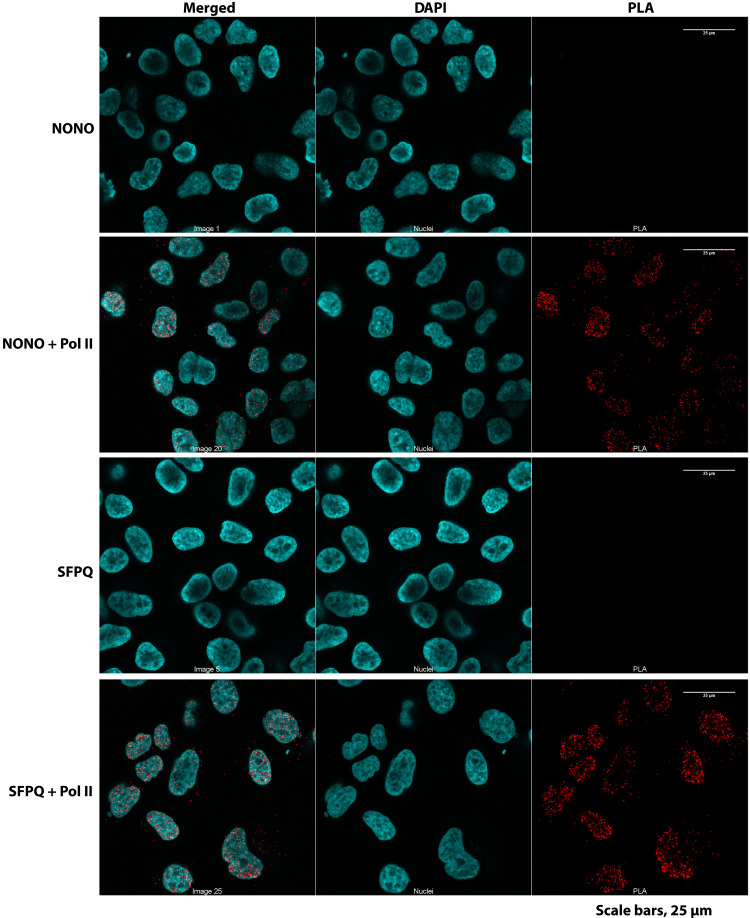
RNA Pol II is closely associated with NONO and SFPQ in vivo. PLAs using anti-NONO, anti-NONO plus anti–Pol II, anti-SFPQ, or anti-SFPQ plus anti–Pol II antibodies on fixed HeLa cells. PLAs using anti-NONO or anti-SFPQ served as a negative control. PLA signal is shown in red; 4′,6-diamidino-2-phenylindole (DAPI) nuclear staining in cyan. Scale bars, 25 μm.

Last, proximity ligation assays (PLAs) showed Pol II, NONO, and SFPQ to be in close proximity to each other in vivo, as predicted from the BMC data (Fig. 7). These data show that besides the core proteins essential for PIC formation and early transcription, BMCs contain proteins known to be necessary for elongation and proteins that may be specialized to promote phase separation in vivo and that tightly colocalize with RNA Pol II.

## DISCUSSION

Biochemistry, in some respects, is hampered by the inability to recapitulate the concentrated environments that exist in a cell. Classical biochemistry uses relatively dilute concentrations of proteins to examine both catalytic and noncatalytic activities. However, the nuclear and cytoplasmic interiors are very dense and gel-like, with protein concentrations in the range of 100 to 400 mg/ml, and this macromolecular crowding imposes unique conditions. At these concentrations, kinetics are non–Michaelis-Menton (*5*, *53*), diffusion is anomalous (*5*), and weak interactions become relevant (*3*, *5*, *12*, *54*, *55*). Some of these features can be recapitulated in vitro, by forcing a phase transition with high recombinant protein concentrations and a volume excluder. However, whether the degree to which condensates are physiological or functional is controversial.

The BMCs herein are functional and capable of PIC formation, transcriptional initiation, and elongation. They reflect the high intranuclear concentrations of proteins and DNA and are the product of at least one active process. The BMCs are visible microscopically and are approximately 0.5 to 1 μm in diameter, and their macromolecules contribute ~100 mg/ml to their density, enabling isolation by low-speed centrifugation (Figs. 1 and 5). A phase transition becomes manifest as promoter DNA is titrated into nuclear extract or vice versa (Fig. 2). There is an increase in both BMC numbers and amount of Alexa Fluor 488 CMV DNA incorporated into the BMCs, which indicates that the DNA is entering the concentrated phase of the BMC, as expected of a phase transition (*33*, *34*). BMCs include ~2 templates indicating little intrinsic proclivity for the CMV promoter to cluster (*56*, *57*).

### BMCs are transcriptionally active

Transcription occurs within the BMC, based on several observations. First, a relatively low centrifugation, as low as 2300*g*, pellets labeled RNA produced in the transcription reactions, except for a subset between +20 and +75 and most of the labeled tRNAs (Figs. 1B and 5A). Maneuvers that disrupted BMCs also disturbed transcription. The addition of KCl to a final concentration of 350 mM or the addition of Sarkosyl allowed further elongation beyond the pause sites and dissolved the BMCs (Fig. 3, A to D).

Second, BMCs treated with a short pulse of ^32^P-CTD and unlabeled GTP, ATP, and UTP were transcriptionally active after removal of the dilute phase and addition of NTPs: The full complement of RNAs was produced, as seen in the standard pulse-chase assays (compare Fig. 1B to Fig. 4B). Third, BMCs isolated by centrifugation, before transcription initiation, were transcriptionally competent while the supernatant was devoid of activity (Fig. 4C). Last, we observed BrU incorporation in the BMCs in the presence of GAC but not with BrU alone (Fig. 4D). We did not observe that the labeled RNA synthesized, isolated, and added back to unlabeled BMCs adhered to the BMCs: exogenously labeled RNA/transcription product remains entirely in the supernatant when added to BMCs (Fig. 4D).

Furthermore, the requirements for transcription in BMCs are more complex than those needed to reconstitute a transcriptionally competent promoter complex from purified components. This is best demonstrated upon the addition of the OGT catalytic inhibitor ST045849, which we previously showed inhibited PIC formation and transcription (*36*, *37*). We show here that the addition of this inhibitor before PIC formation both abolishes transcription in a pulse-chase assay (Fig. 4E) and concomitantly inhibits formation of BMCs (Fig. 4F). Consistent with the role of OGT in BMC formation, OGT was detected in the BMCs (Fig. 6G). The formation of a functional BMC is an active process, one that requires catalysis of uridine diphosphate-N-acetylglucosamine (UDP-GlcNAc) and the GlcNAcylation of protein(s) in the extract during PIC formation. These results show that transcription occurs within the BMCs and that any physical disruption of the BMC disrupts either proper elongation or transcription altogether.

### The transcriptionally active BMC is an extension of a PIC

Our data show that transcriptionally active BMCs form in a complex heterogeneous protein environment outside of the cell. Given that transcription initiation and elongation occur in the BMCs, the BMCs appear to be a self-contained environment. We propose a model in which the promoter DNA acts as a scaffold, nucleating the assembly of a BMC via formation of a PIC and the subsequent recruitment of additional machinery and factors. The proteins in the PIC contain intrinsically disordered regions such as the RNA Pol II CTD (*58*, *59*) and recruit other proteins, such as RNA binding proteins NONO, SFPQ, FUS, and elongation factors, to form a gel- or lattice-like interlocking protein environment surrounding the promoter. It is through a multitude of weak interactions coupled to the much stronger interactions of the general transcriptional machinery and promoter DNA that the full complement of transcriptional machinery assembles. The BMCs forms from a nuclear extract, where each protein species is effectively far below its concentration to phase transition on its own; the final protein concentration of the nuclear extract in the in vitro transcription assay is approximately 2 mg/ml. In contrast, other types of condensates formed in vitro used recombinant protein at very high concentrations along with volume excluders such as polyethylene glycol or dextran.

We hypothesize that the BMCs are a manifestation of the emergent biochemical properties of the transcription machinery and DNA that occurs in the nucleus: formation of a dense environment of protein-protein and protein-DNA interactions that recapitulates RNA Pol II initiation, pausing, termination, and elongation. BMCs represent a transition from the submicroscopic, molecular scale to a microscopic scale of assembly to form spatially organized, self-contained biochemical reaction vessels, a self-organization of biological matter on a scale far larger than that of the promoter and a classic PIC. The transcriptional and the nuclear environment in general should be considered a complex combination of strong and weak interactions between proteins and DNA. It is this combination of strong and weak interactions that accurately recapitulate RNA Pol II transcriptional regulation. The BMCs described herein are a manifestation of the intrinsic ability of protein and promoter DNA polymers, to sustain numerous multivalent weak interactions in vivo.

## MATERIALS AND METHODS

### In vitro transcription assays

In vitro transcription assays were as described previously (*29*). Briefly, final transcription conditions were 10 mM Hepes (pH 7.5), 50 mM KCl, 6.25 mM MgCl_2_, 5% glycerol, 0.2 mM EDTA, ~40 μg of HeLa nuclear extract (nuclear extracts were centrifuged for 5 min at 21,000*g* at 4°C to remove any insoluble materials before using them in either pulse-chase or microscopy experiments), and 200 ng of pGL2-CMV IE promoter DNA linearized with Eco RI (*28*), in a final volume of 25 μl. PICs were formed at 25°C for 30 min. All PICs/BMCs were assembled with 0.2 pmol of DNA and 40 μg nuclear extract unless otherwise noted. A 30 s pulse was started with the addition of 0.5-μCi ^32^P-α-CTP (3000 Ci/mmol; PerkinElmer) and 0.5 mM guanine/uridine/adenosine triphosphate. A 5 min chase was done by adding cold CTP to 3 mM final concentration. Reactions were centrifuged as indicated, and then, pellet and supernatants were processed by the addition of 220 μl of stop buffer, phenol/chloroform extraction, and ethanol precipitation to isolate the labeled RNA. Samples were then analyzed by electrophoresis on 10% TBE/urea/polyacrylamide gels followed by autoradiography (*26*, *27*).

KCl (350 mM final concentration) or Sarkosyl (0.6% final concentration) was added after the 30 s pulse followed immediately by the cold CTP chase. For the OGT inhibition, either 0.5 μl of dimethyl sulfoxide or 0.5 μl of 10 mM ST045849 (Tocris) was added to the transcription reaction without DNA for 10 min, followed by the addition of CMV promoter DNA for 30 min. This was followed by the standard 30 s pulse/5 min chase and subsequent RNA isolation, TBE/urea polyacrylamide electrophoresis, and autoradiography.

Nuclear extracts were made from HeLa cells supplied by Cell Culture Company (Minneapolis, MN). Briefly, extracts were made with a protocol from Workman and colleagues and which the nuclear extraction was done using 0.4 M KCl (*60*) and dialyzed versus HEGK100 [20 mM Hepes (pH 7.6), 100 mM KCl, 20% glycerol, 0.2 mM EDTA, and 0.25 mM dithiothreitol].

Sucrose cushions were made in 10 mM Hepes (pH 7.6), 50 mM KCl, 0.1 mM EDTA, 5% glycerol, and the indicated amount (w/v) of sucrose. Five hundred microliters of each cushion was placed in an Eppendorf tube, and the transcription reaction was gently pipetted onto the top of the cushion. Cushions were centrifuged for 5 min at 21,000*g* at room temperature. Labeled RNA was then processed and analyzed by TBE/urea polyacrylamide electrophoresis and autoradiography. The density approximation was obtained by subtracting the density of water (1 mg/ml) from the density of 25% sucrose (1.1 mg/ml).

Pulsed-stage BMCs were made by doing a 30 s pulse and isolated by centrifugation 5 min at 21,000*g*, washed briefly with 100 μl of transcription buffer [20 mM Hepes (pH 7.6), 60 mM KCl, 3 mM MgCl_2_, and bovine serum albumin (BSA) (200 μg/ml)], and suspended in 20 μl of transcription buffer. NTPs were added to 0.5 mM final concentration for 5 min to chase-labeled RNAs into longer products. PIC-stage BMCs were treated in the same manner, except that the centrifugation was done before the pulse. Labeled RNA was then processed and analyzed by TBE/urea polyacrylamide electrophoresis and autoradiography.

### Microscopy

To construct the Alexa Fluor 488 and Alexa Fluor 594 CMV promoter DNA, PCR of pGL2-CMV was done using Alexa Fluor 488 (or 594)-GTA CTG TAA CTG AGC TAA CAT AA and CGA CTG AAA TCC CTG GTA ATC CGT T primers to amplify an approximately 1.3-kb DNA product (*31*).

Imaging was done using a Zeiss LSM 780 or 880 confocal microscope with a 63× objective. Alexa Fluor 488–CMV (0.2 pmol) was mixed with NE as above for the in vitro transcription reactions. After 30 min, 4 μl of an in vitro transcription reaction was placed on a coverslip (Electron Microscopy Sciences, #72291-09) surrounded by a rubber gasket (double-sided adhesive, Grace Biolabs, JTR12R-A-0.5), and then, the slide was placed on top. Antibodies were added just before adding the transcription reaction to the coverslip. One microliter of a 1:10 dilution of Alexa Fluor 594–conjugated CTD antibody 8WG16 was used to detect RNA Pol II. For antibody immunostaining, 1 μl of a 1:10 dilution of a primary antibody (0.2 mg/ml stock) was added to 10 μl of PIC stage BMCs followed by 1 μl of 1:100 dilution of Alexa Fluor 488 anti-rabbit secondary antibody (2 mg/ml stock). Four microliters of this mixture was then applied to a coverslip as above.

For the BrU labeling, we formed BMCs with CMV plasmid DNA, added either BrUTP (as a negative control) or BrUTP + ATP, GTP, and CTP (to initiate transcription) (all at 0.5 mM final concentration) for 5 min, fixed the BMCs with 2% paraformaldehyde (final concentration), added 5 μl of the sample on a coverslip (poly-l-lysine coated, Electron Microscopy Sciences, #72292-16), and allowed them to dry overnight in the dark. The coverslips were incubated 15 min in Tris-buffered saline/0.05% Tween 20 (TBST) and 45 min in TBST/3% BSA and carefully dried around the dried sample. One hundred microliters of 1/1000 anti-BrU antibody in TBST/3% BSA was added for 60 min, rinsed 15 min in TBST, followed by adding 100 μl of 1:2000 anti-rabbit Alexa Fluor 488 secondary in TBST and washing in TBST 15 min. Coverslip was dried with a Kimwipe around the sample, and a silicone rubber gasket was applied, followed by 5 μl of PBS and attaching the coverslip to a slide, followed by imaging.

### Image analysis

Fiji/ImageJ was used for microscopy image analysis, processing, and quantitations. R statistical software (4.1.2) was used for data analysis. Images were adjusted to visualize BMCs. A duplicate channel image was made and subjected to Gaussian blur filtering. Background was then eliminated by manually setting the threshold to where BMCs were included. This mask was used to quantitate the original image (*61*). Quantitations using Fiji included mean gray value, area, intensity density, and raw intensity density. A proxy for the actual partition coefficient was calculated by averaging the sum of the mean gray value (pixels) per BMC and multiplying that by the average number of BMCs for either Alexa Fluor 488–CMV or Alexa Fluor 594 RNA Pol II antibody in three separate images. Center of mass calculations were done using the JaCoP module in Fiji (*62*).

### In situ PLA

PLA was carried out using the Duolink In Situ PLA Kit (Sigma-Millipore) following the manufacturer’s protocol. Briefly, HeLa cells (1 × 10^4^) were plated on ibidi 15 μ-slide angiogenesis slide (#81506) for 1 hour and used for PLA as described by the manufacturer’s protocol. The following primary antibodies used for PLA: anti-NONO rabbit polyclonal antibody (1:100), anti-SFPQ rabbit polyclonal antibody (1:100), and anti-RNA Pol II CTD (1:100). Examination of cells was performed with a Zeiss LSM 880 multiphoton confocal microscope. Prominent red PLA dots were indicative of close association of two proteins of interest.

### Antibodies

Alexa Fluor 594 Pol II Antibody 8WG16 (Santa Cruz Biotechnology, sc-56767 AF594), Pol II antibody F12 (Alexa Fluor 488, Santa Cruz Biotechnology, sc-55492 AF488; Alexa Fluor 594, Santa Cruz Biotechnology, sc-55492 AF594), NONO (anti-nmnt/p54, Abcam, ab70335), SFPQ (Abcam, ab177149), anti-BrU antibody (Abcam, ab152095), CDK9 antibody (Bethyl A303-493A), FUS antibody (Abcam, ab23439), PARP-1 antibody (Abcam, ab14459), Leo1 antibody (Bethyl, A300-175A), HCF-1 antibody (Bethyl, A301-399A), OGT (ABclonal, A1990), PSPC-1 (Abcam, ab104238), FUS (Abcam, ab23439), Alexa Fluor 488 secondary anti-rabbit antibody (Invitrogen, A11008), and Alexa Fluor 488 secondary anti-mouse antibody (Invitrogen, A11001).
