# Supplementary material for: Self-assembly of promoter DNA and RNA Pol II machinery into transcriptionally active biomolecular condensates
Source: Sci Adv. 2023 Oct 18;9(42):eadi4565. doi: 10.1126/sciadv.adi4565 (PMC10584347; doi:10.1126/sciadv.adi4565)
Supplement: Supplementary file 1 — Figs. S1 to S3 [file sciadv.adi4565_sm.pdf]

Science Advances
AAAS

Supplementary Materials for

Self-assembly of promoter DNA and RNA Pol II machinery into transcriptionally active biomolecular condensates

Brian A. Lewis *et al.*

Corresponding author: Brian A. Lewis, brian.lewis@nih.gov; David Levens, levensd@mail.nih.gov

Sci. Adv. **9**, eadi4565 (2023)
DOI: 10.1126/sciadv.adi4565

This PDF file includes:

Figs. S1 to S3

Supplemental Figures

Fig. S1. Control transcription assays for pulse-chase experiments.
A. Lane 1 shows a standard pulse-chase transcription. Lane 2 shows the labeling of the tRNAs in the nuclear extract in the absence of promoter DNA. Lane 3 shows that 2 μg/mL α-amanitin blocks RNA pol II transcription from the CMV promoter (*32*). B. Shown is a shorter exposure of the spin experiment seen in Figure 1. C. Shown is a titration of the pGL2 vector only DNA in lanes 1-3 (0.05, 0.1, and 0.2 μg DNA, respectively) compared to 0.2 μg CMV promoter DNA (in pGL2).

Fig. S2. DNA and NE titrations show functional concentration transitions.
A. CMV promoter DNA titration related to Fig. 2ABC. Here in vitro transcriptions were done as in Fig. 1. DNA was titrated into 40 μg NE. B. NE Titration related to Fig. 2DEF. In vitro transcriptions were as in Fig. 1. Here, 0.2 pmol CMV promoter DNA was incubated with the indicated amount of nuclear extract followed by a pulse-chase assay.

Fig. S3. RNA pol II levels follow BMC area.
A. and B. Plot of RNA pol II intensities v. BMC area, related to Fig. 2G. The Alexa 488- or Alexa-594 F12 antibody recognizes the N-terminus of the large subunit of RNA pol II. PICs were assembled with 5 μL NE and 0.2 pmol CMV promoter DNA for 30’ at RT, followed by visualization of RNA pol II with the F12 antibody. Intensities and area were then calculated using Fiji and the data plotted using R software. C. Laser scanning confocal microscopy of F12 anti-pol II images.

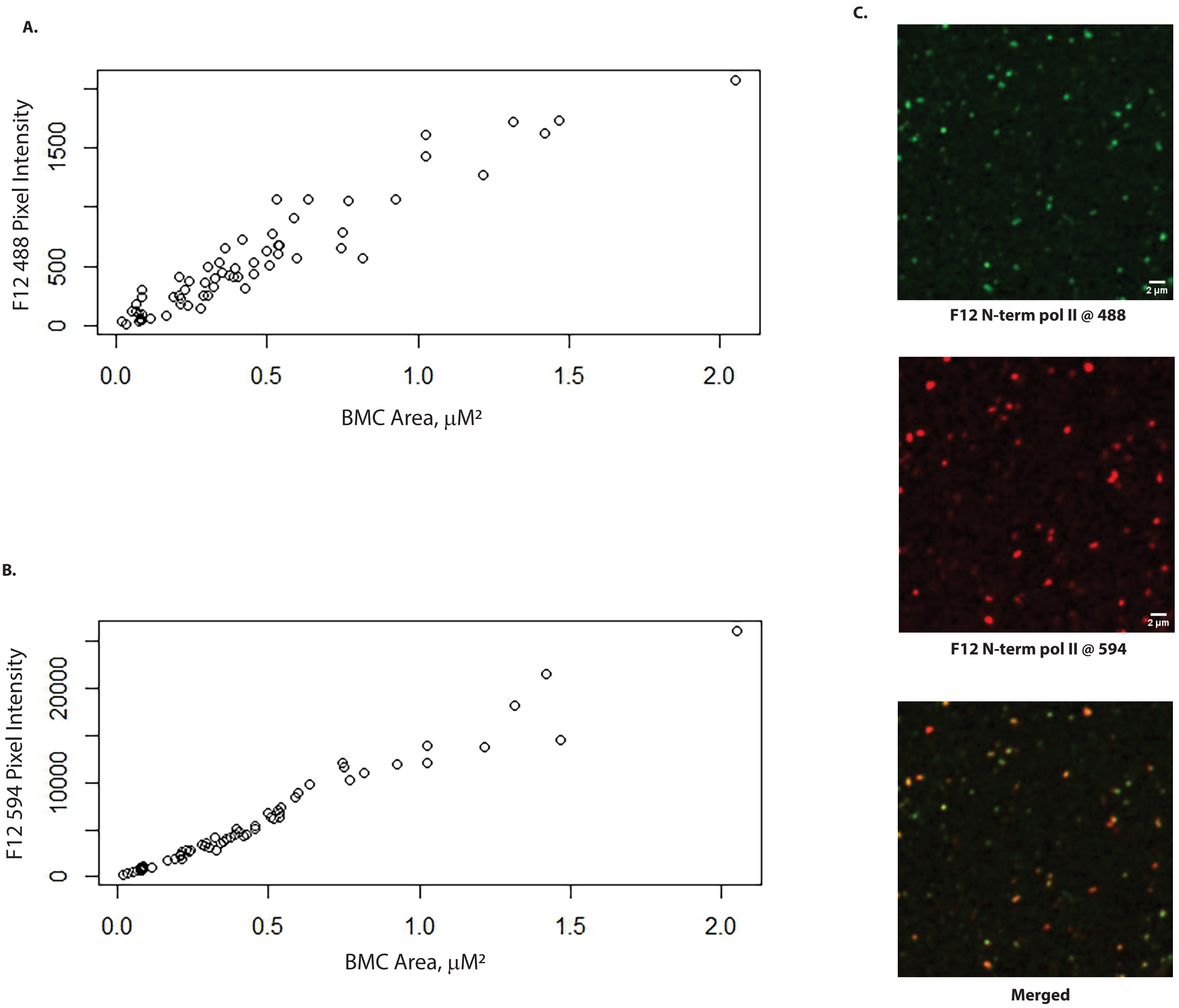
A.
F12 488 Pixel Intensity
0
500
1500
0.0
0.5
1.0
1.5
2.0
BMC Area, µM²
B.
F12 594 Pixel Intensity
0
10000
20000
0.0
0.5
1.0
1.5
2.0
BMC Area, µM²
C.
2 µm
F12 N-term pol II @ 488
2 µm
F12 N-term pol II @ 594
Merged

Fig. S3

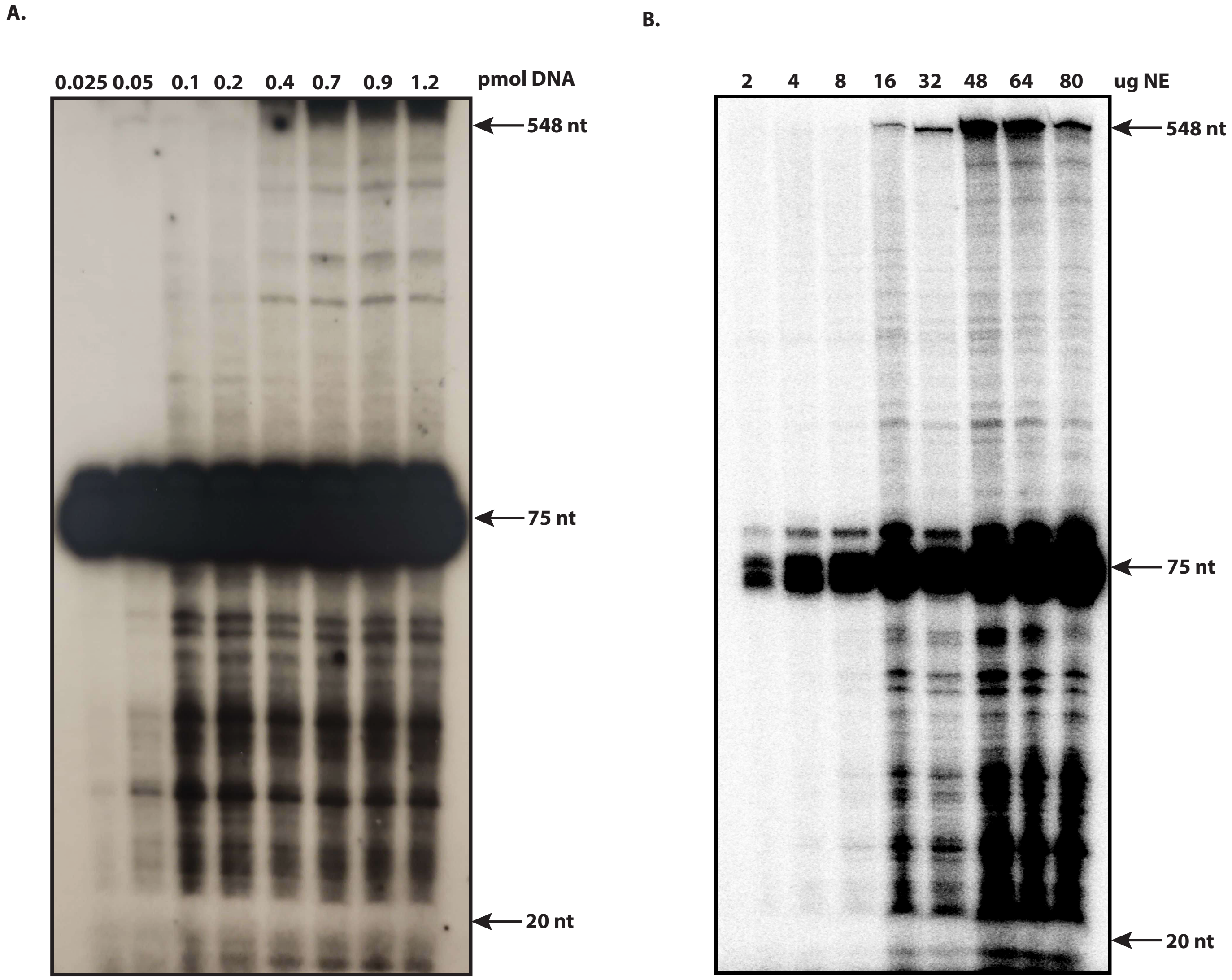
A.
0.025 0.05 0.1 0.2 0.4 0.7 0.9 1.2
pmol DNA
548 nt
75 nt
20 nt
B.
2 4 8 16 32 48 64 80
ug NE
548 nt
75 nt
20 nt

Fig. S2

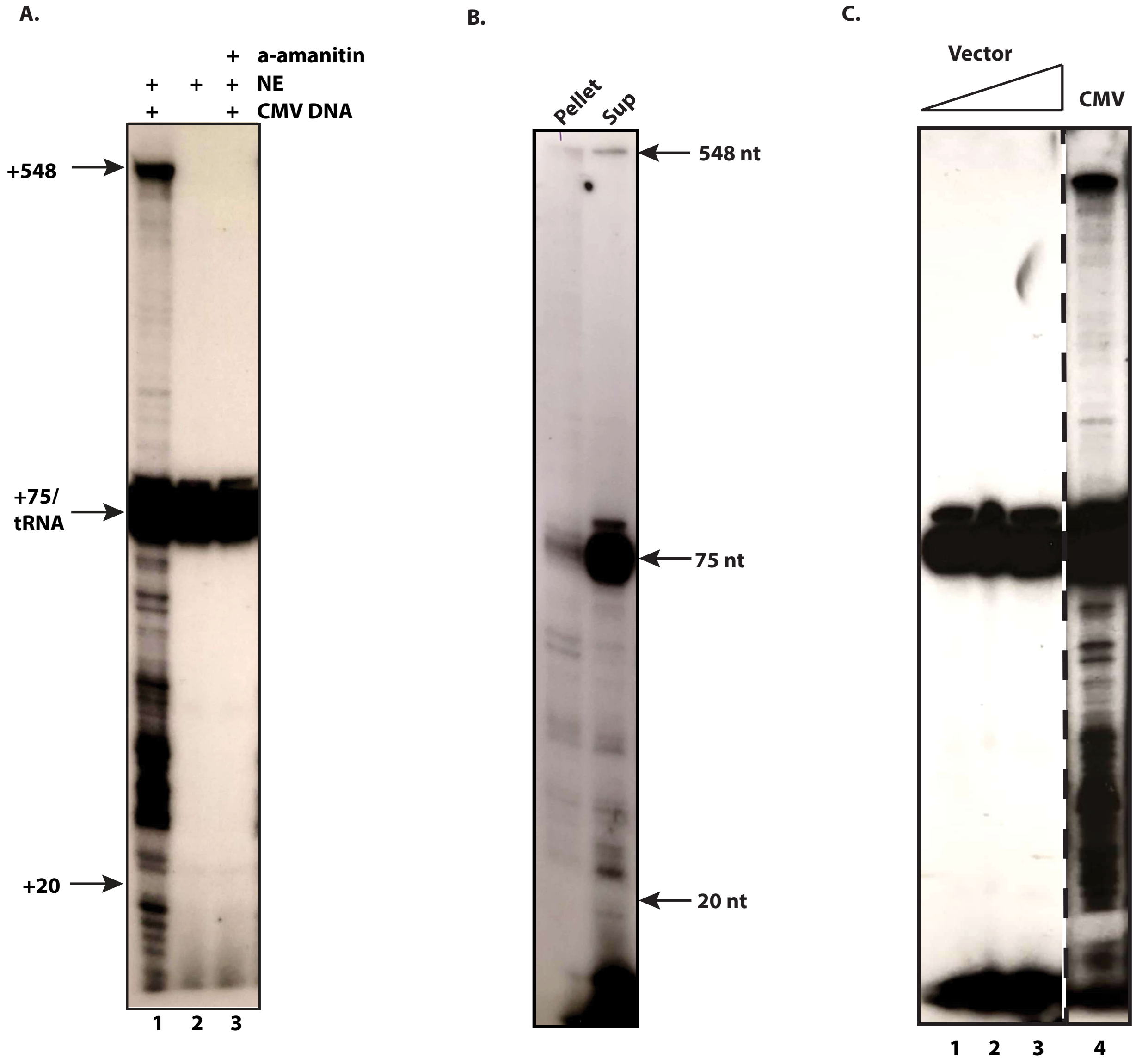
A.
+ a-amanitin
+ + + NE
+ + CMV DNA
+548
+75/ tRNA
+20
1 2 3
B.
Pellet Sup
548 nt
75 nt
20 nt
C.
Vector
CMV
1 2 3 4

Fig. S1
